# Quantitative multimodal multiparametric imaging in Alzheimer’s disease

**DOI:** 10.1007/s40708-015-0028-9

**Published:** 2016-01-08

**Authors:** Qian Zhao, Xueqi Chen, Yun Zhou

**Affiliations:** 1 Department of Nuclear Medicine, General Hospital of Ningxia Medical University, Yinchuan, 750004 China; 2 The Russell H. Morgan Department of Radiology and Radiological Science, Johns Hopkins University School of Medicine, Baltimore, MD 21287 USA; 3 Department of Nuclear Medicine, Peking University First Hospital, Beijing, 100034 China

**Keywords:** Alzheimer’s disease, Neuroimaging, PET, MRI, Amyloid beta, Multimodal

## Abstract

Alzheimer’s disease (AD) is a progressive neurodegenerative disorder, causing changes in memory, thinking, and other dysfunction of brain functions. More and more people are suffering from the disease. Early neuroimaging techniques of AD are needed to develop. This review provides a preliminary summary of the various neuroimaging techniques that have been explored for in vivo imaging of AD. Recent advances in magnetic resonance (MR) techniques, such as functional MR imaging (fMRI) and diffusion MRI, give opportunities to display not only anatomy and atrophy of the medial temporal lobe, but also at microstructural alterations or perfusion disturbance within the AD lesions. Positron emission tomography (PET) imaging has become the subject of intense research for the diagnosis and facilitation of drug development of AD in both animal models and human trials due to its non-invasive and translational characteristic. Fluorodeoxyglucose (FDG) PET and amyloid PET are applied in clinics and research departments. Amyloid beta (Aβ) imaging using PET has been recognized as one of the most important methods for the early diagnosis of AD, and numerous candidate compounds have been tested for Aβ imaging. Besides in vivo imaging method, a lot of ex vivo modalities are being used in the AD researches. Multiphoton laser scanning microscopy, neuroimaging of metals, and several metal bioimaging methods are also mentioned here. More and more multimodality and multiparametric neuroimaging techniques should improve our understanding of brain function and open new insights into the pathophysiology of AD. We expect exciting results will emerge from new neuroimaging applications that will provide scientific and medical benefits.

## Introduction

Alzheimer’s disease (AD) is a progressive neurodegenerative disorder that gradually destroys brain cells, causing changes in memory, thinking, and other dysfunction of brain functions [1]. AD is considered to a prolonged preclinical stage where neuropathological changes precede the clinical symptoms [2]. An estimation of 35 million people worldwide is living with this disease. If effective treatments are not discovered in a timely fashion, the number of AD cases is anticipated to rise to 113 million by 2050 [3].

Amyloid beta (Aβ) and tau are two of the major biomarkers of AD, and have important and different roles in association with the progression of AD pathophysiology. Jack et al. established hypothetical models of the major biomarkers of AD. By renewing and modifying the models, they found that the two major proteinopathies underlying AD biomarker changes, Aβ and tau, may be initiated independently in late onset AD where they hypothesize that an incident Aβ pathophysiology can accelerate an antecedent limbic and brainstem tauopathy [4]. MRI technique was used in the article, which revealed that the level of Aβ load was associated with a shorter time-to-progression of AD [5]. This warrants an urgent need to develop early neuroimaging techniques of AD neuropathology that can detect and predict the disease before the onset of dementia, monitor therapeutic efficacy in halting and slowing down progression in the earlier stage of the disease.

There have been various reports on the imaging assessments of AD. Some measurements reflect the pathology of AD directly, including positron emission tomography (PET) amyloid imaging and cerebrospinal fluid (CSF) beta-amyloid 42 (Aβ42), while others reflect neuronal injury associated with AD indirectly, including CSF tau (total and phosphorylated tau), fluorodeoxy-d-glucose (FDG)-PET, and MRI. AD Neuroimaging Initiative (ADNI) has been to establish the optimal panel of clinical assessments, MRI and PET imaging measures, as well as other biomarkers from blood and CSF, to inform clinical trial design for AD therapeutic development. At the same time, it has been highly productive in generating a wealth of data for elucidating disease mechanisms occurring during early stages of preclinical and prodromal AD [6].

Single neuroimaging often reflects limit information of AD. As a result, multimodal neuroimaging is widely used in neuroscience researches, as it overcomes the limitations of individual modalities. Multimodal multiparametric imaging mean the combination of different imaging techniques, such as PET, MRI, simultaneously or separately. The multimodal multiparametric imaging enables the visualization and quantitative analysis of the alterations in brain structure and function, such as PET/CT, and PET/MRI. [7]. In this review article, we summarize and discuss the main applications, findings, perspectives as well as advantages and challenges of different neuroimaging in AD, especially MRI and PET imaging.

## Magnetic resonance imaging

MRI demonstrates specific volume loss or cortical atrophy patterns with disease progression in AD patients [8–10]. There are several MRI techniques and analysis methods used in clinical and scientific research of AD. Recent advances in MR techniques, such as functional MRI (fMRI) and diffusion MRI, depict not only anatomy and atrophy of the medial temporal lobe (MTL), but also microstructural alterations or perfusion disturbance within this region.

### Functional MRI

Because of the cognitive reserve (CR), the relationship between severity of AD patients’ brain damage and corresponding clinical symptoms is not always paralleled [11, 12]. Recently, resting-state fMRI (RS-fMRI) is popular for its ability to map brain functional connectivity non-invasively [13]. By using RS-fMRI, Bozzali et al. reported that the CR played a role in modulating the effect of AD pathology on default mode network functional connectivity, which account for the variable clinical symptoms of AD [14]. Moreover, AD patients with higher educated experience were able to recruit compensatory neural mechanisms, which can be measured using RS-fMRI. Arterial spin-labeled (ASL) MRI is another functional brain imaging modality, which measures cerebral blood flow (CBF) by magnetically labeled arterial blood water following through the carotid and vertebral arteries as an endogenous contrast medium. Several studies have concluded the characteristics of CBF changes in AD patients using ASL-MRI [15–17].

At some point in time, sufficient brain damage accumulates to result in cognitive symptoms and impairment. Mild cognitive impairment (MCI) is a condition in which subjects are usually only mildly impaired in memory with relative preservation of other cognitive domains and functional activities and do not meet the criteria for dementia [18], or as the prodromal state AD [19]. MCI patients are at a higher risk of developing AD and up to 15 % convert to AD per year [18]. Binnewijzend et al. have reported the pseudocontinuous ASL could distinguish both MCI and AD from healthy controls, and be used in the early diagnosis of AD [20]. In their continuous study, they used quantitative whole brain pseudocontinuous ASL to compare regional CBF (rCBF) distribution patterns in different types of dementia, and concluded that ASL-MRI could be a non-invasive and easily accessible alternative to FDG-PET imaging in the assessment of CBF of AD patients [21].

### Structure MRI

Structural MRI (sMRI) has already been a reliable imaging method in the clinical diagnosis of AD, characterized as gray matter reduction and ventricular enlargement in standard T1-weighted sequences [9]. Locus coeruleus (LC) and substantia nigra (SN) degeneration was seen in AD. By using new quantitative calculating method, Chen et al. presented a new quantitative neuromelanin MRI approach for simultaneous measurement of locus LC and SN of brainstem in living human subjects [22]. The approach they used demonstrated advantages in image acquisition, pre-processing, and quantitative analysis. Numerous transgenic animal models of amyloidosis are available, which can manipulate a lot of neuropathological features of AD progression from the deposition of β-amyloid [23]. Braakman et al. demonstrated the dynamics of amyloid plaque formation and development in a serial MRI study in a transgenic mouse model [24]. Increased iron accumulation in gray matter is frequently observed in AD. Because of the paramagnetic nature of iron, MRI shows nice potential in the investigating iron levels in AD [25]. Quantitative MRI was shown high sensitivity and specificity in mapping cerebral iron deposition, and helped in the research on AD diagnosis [26].

The imaging patterns are always associated with the pathologic changes, such as specific protein markers. Spencer et al. manifested the relationship between quantitative T1 and T2 relaxation time changes and three immunohistochemical markers: β-amyloid, neuron-specific nuclear protein (a marker of neuronal cell load), and myelin basic protein (a marker of myelin load) in AD transgenic mice [27].

High-field MRI has been successfully applied to imaging plaques in transgenic mice for over a decade without contrast agents [24, 28–30]. Sillerud et al. devised a method using blood–brain barrier penetrating, amyloid-targeted, superparamagnetic iron oxide nanoparticles (SPIONs) for better imaging of amyloid plaque [31]. Then, they successfully used this SPION-MRI to assess the drug efficacy on the 3D distribution of Aβ plaques in transgenic AD mouse [32].

### Diffusion MRI

Diffusion-weighted imaging (DWI) is a sensitive tool that allows quantifying of physiologic alterations in water diffusion, which result from microscopic structural changes.

Diffusion tensor imaging (DTI) is a well-established and commonly employed diffusion MRI technique in clinical and research on neuroimaging studies, which is based on a Gaussian model of diffusion processes [33]. In general, AD is associated with widespread reduced fractional anisotropy (FA) and increased mean diffusivity (MD) in several regions, most prominently in the frontal and temporal lobes, and along the cingulum, corpus callosum, uncinate fasciculus, superior longitudinal fasciculus, and MTL-associated tracts than healthy controls [34–37]. Acosta-Cabronero et al. reported increased axial diffusivity and MD in the splenium, which were the earliest abnormalities in AD [38]. FA and radial diffusivity (DR) differences in the corpus callosum, cingulum, and fornix were found to separate individuals with MCI who converted to AD from non-converters [39]. DTI was also found to be a better predictor of AD-specific MTL atrophy when compared to CSF biomarkers [40]. These findings suggested the potential clinical utility of DTI as early biomarkers of AD and its progression. However, an increase in MD and DR and a decrease in FA with advancing age in selective brain regions have been previously reported [41, 42]. Diffusion MRI can be also used in the classifying of various stages of AD. Multimodal classification method, which combined fMRI and DTI, separated more MCI from healthy controls than single approaches [43].

In recent years, tau has emerged as a potential target for therapeutic intervention. Tau plays a critical role in the neurodegenerative process forming neurofibrillary tangles, which is a major hallmark of AD and correlates with clinical disease progression. Wells et al. applied multiparametric MRI, containing high-resolution structure MRI (sMRI), a novel chemical exchange saturation transfer (CEST) MRI, DTI, and ASL, and glucose CEST to measure changes of tau pathology in AD transgenic mouse [44].

Besides DWI MRI, perfusion-weighted imaging (PWI) is another advanced MR technique, which could measure the cerebral hemodynamics at the capillary level. Zimny et al. evaluated the correlation of MTL with both DWI and PWI in AD and MCI patients [45].

## Positron emission tomography

PET is a specific imaging technique applying in researches of brain function and neurochemistry of small animals, medium-sized animals, and human subjects [46–48]. As a particular brain imaging technique, PET imaging has become the subject of intense research for the diagnosis and facilitation of drug development of AD in both animal models and human trials due to its non-invasive and translational characteristic. PET with various radiotracers is considered as a standard non-invasive quantitative imaging technique to measure CBF, glucose metabolism, and β-amyloid and tau deposition.

### FDG-PET

To date, 18F-FDG is one of the best and widely used neuroimaging tracers of PET, which employed for research and clinical assessment of AD [49]. Typical lower FDG metabolism was shown in the precuneus, posterior cingulate, and temporal and parietal cortex with progression to whole brain reductions with increasing disease progress in AD brains [50, 51]. FDG-PET imaging reflects the cerebral glucose metabolism, neuronal injury, which provides indirect evidence on cognitive function and progression that cannot be provided by amyloid PET imaging.

Schraml et al. [52] identified a significant association between hypometabolic convergence index and phenotypes using ADNI data. Some researchers also used 18F-FDG-PET to analyze genetic information with multiple biomarkers to classify AD status, predicting cognitive decline or MCI to AD conversion [53–55]. Trzepacz et al. [56] reported multimodal AD neuroimaging study, using MRI, 11C-PiB PET, and 18F-FDG-PET imaging to predict MCI conversion to AD along with APOE genotype. Zhang et al. [57] compared the genetic modality single-nucleotide polymorphism (SNP) with sMRI, 18F-FDG-PET, and CSF biomarkers, which were used to differentiate healthy control, MCI, and AD. They found FDG-PET is the best modality in terms of accuracy.

### Amyloid beta PET

Aβ, the primary constituent of senile plaques, and tau tangles are hypothesized to play a primary role in the pathogenesis of AD, but it is still hard to identify the fundamental mechanisms [58–60]. Aβ plaque in brain is one of the pathological hallmarks of AD [61, 62]. Accumulation of Aβ peptide in the cerebral cortex is considered one cause of dementia in AD [63]. Numerous studies have involved in vivo PET imaging assessing cortical β-amyloid burden [64–66].

Aβ imaging using PET has been recognized as one of the most important methods for the early diagnosis of AD [67]. Numerous candidate compounds have been tested for Aβ imaging, such as 11C-PiB [68], 18F-FDDNP [69], 11C-SB-13 [70], 18F-BAY94-9172 [71], 18F-AV-45 [72], 18F-flutemetamol [73, 74], 11C-AZD2184 [75], and 18F-ADZ4694 [76], 11C-BF227 and 18F-FACT [77].

Several amyloid PET studies examined genotypes, phenotypes, or gene–gene interactions. Ramanan et al. [78] reported the GWAS results with 18F-AV-45 reflecting the cerebral amyloid metabolism in AD for the first time. Swaminathan et al. [79] revealed the association between plasma Aβ from peripheral blood and cortical amyloid deposition on 11C-PiB. Hohman et al. [80] reported the relationship between SNPs involved in amyloid and tau pathophysiology with 18F-AV-45 PET.

Among the PET tracers, 11C-PiB, which has a high affinity for fibrillar Aβ, is a reliable biomarker of underlying AD pathology [68, 81]. It shows cortical uptake well paralleled with AD pathology [82, 83], has recently been approved for use by the Food and Drug Administration (FDA, April 2012) and the European Medicines Agency (January 2013). 18F-GE-067 (flutemetamol) and 18F-BAY94-9172 (florbetaben) have also been approved by the US FDA in the last 2 years [84, 85].

18F-Florbetapir (also known as 18F-AV-45) exhibits high affinity specific binding to amyloid plaques. 18F-AV-45 labels Aβ plaques in sections from patients with pathologically confirmed AD [72].

It was reported in several research groups that 18F-AV-45 PET imaging showed a reliability of both qualitative and quantitative assessments in AD patients, and Aβ+ increased with diagnostic category (healthy control < MCI < AD) [82, 86, 87]. Johnson et al. used 18F-AV-45 PET imaging to evaluate the amyloid deposition in both MCI and AD patients qualitatively and quantitatively, and found that amyloid burden increased with diagnostic category (MCI < AD), age, and APOEε4 carrier status [88]. Payoux et al. reported the equivocal amyloid PET scans using 18F-AV-45 associated with a specific pattern of clinical signs in a large population of non-demented older adults more than 70 years old [89].

More and more researchers consider combination and comparison of multiple PET tracers targeting amyloid plaque imaging together. Bruck et al. compared the prognostic ability of 11C-PiB PET, 18F-FDG-PET, and quantitative hippocampal volumes measured with MR imaging in predicting MCI to AD conversion. They found that the FDG-PET and 11C-PiB PET imaging are better in predicting MCI to AD conversion [90]. Hatashita et al. used 11C-PiB and FDG-PET imaging to identify MCI due to AD, 11C-PiB showed a higher sensitivity of 96.6 %, and FDG-PET added diagnostic value in predicting AD over a short period [91].

Besides, new Aβ imaging agents were radiosynthesized. Yousefi et al. radiosynthesized a new Aβ imaging agent 18F-FIBT, and compared the three different Aβ-targeted radiopharmaceuticals for PET imaging, including 18F-FIBT, 18F-florbetaben, and 11C-PiB [92]. 11C-AZD2184 is another new PET tracer developed for amyloid senile plaque imaging, and the kinetic behavior of 11C-AZD2184 is suitable for quantitative analysis and can be used in clinical examination without input function [75, 93, 94].

## Multimodality imaging: PET/MRI

Several diagnostic techniques, including MRI and PET, are employed for the diagnosis and monitoring of AD [95]. Multimodal imaging could provide more information in the formation and key molecular event of AD than single method. It drives the progression of neuroimaging research due to the recognition of the clinical benefits of multimodal data [96], and the better access to hybrid devices, such as PET/MRI [97].

Maier et al. evaluated the dynamics of 11C-PiB PET, 15O-H_2_O-PET, and ASL-MRI in transgenic AD mice and concluded that the AD-related decline of rCBF was caused by the cerebral Aβ angiopathy [98]. Edison et al. systematically compared 11C-PiB PET and MRI in AD, MCI patients, and controls. They thought that 11C-PiB PET was adequate for clinical diagnostic purpose, while MRI remained more appropriate for clinical research [99]. Zhou et al. investigated the interactions between multimodal PET/MRI in elder patients with MCI, AD, and healthy controls, and confirmed the invaluable application of amyloid PET and MRI in early diagnosis of AD [100]. Kim et al. reported that Aβ-weighted cortical thickness, which incorporates data from both MRI and amyloid PET imaging, is a consistent and objective imaging biomarker in AD [101].

## Other imaging modalities

Multiphoton non-linear optical microscope imaging systems using ultrafast lasers have powerful advantages such as label-free detection, deep penetration of thick samples, high sensitivity, subcellular spatial resolution, 3D optical sectioning, chemical specificity, and minimum sample destruction [102, 103]. Coherent anti-Stokes–Raman scattering (CARS), two-photon excited fluorescence (TPEF), and second-harmonic generation (SHG) microscopy are the most widely used biomedical imaging techniques [104–106].

Some researchers have reported in vivo imaging of senile plaque and collagen using multiphoton laser scanning microscopy or auto-fluorescence and SHG images in AD mouse model [107, 108]. Lee et al. developed a multimodal multiphoton non-linear optical microspectroscopy imaging system based on a small-diameter probe with gradient-index lenses combing CARS, TPEF, and SHG into one platform for imaging distinct molecular structures and components of brain tissue associated with AD transformation [109].

Metal dyshomeostasis is frequently observed in AD due to anomalous binding of metals such as iron (Fe), copper (Cu), and zinc (Zn), or impaired regulation of redox-active metals inducing the neuronal damage. Neuroimaging of metals in a variety of intact brain cells and tissues is emerging as an important tool for increasing our understanding of the role of metal dysregulation in AD. Braidy et al. reviewed the metal bioimaging in AD [110]. Several imaging techniques, such as laser ablation inductively coupled mass spectrometry (MS), X-ray fluorescence microscopy, MALDI imaging mass spectrometry (MALDI-MS), and Fourier transform infrared spectroscopy,, have been used to study in AD [111–114]. Several limitations of metal bioimaging, such as lower spatial resolution and detection sensitivity, make it not as valuable as PET and MRI imaging.

## Perspective of neuroimaging in precision imaging

Precision medicine is a phrase that is often used to describe how genetic information about a person’s disease is being used to diagnose or treat their disease. Understanding the genetic changes that are in AD formation is leading to more effective treatment strategies. The prospect of a personalized or precision medicine for AD, and for its incorporation in therapeutic trial design, is predicated on the ability to use an individual’s genetic profile to refine predisposition to disease, characteristics such as likely rate of progression, and predicted therapeutic and side effect responses to various therapeutic strategies [115]. At present, the genetic data associated with AD clinical drug development were mainly focused on APOE, which was commonly used as stratification factor or covariate to adjust for heterogeneity [116, 117].

Ultimately, imaging-genetic endophenotype studies may provide a link between genetics and disease topography by elucidating those areas of the brain most associated with known and potential pathological genotypes. APOE genotype may be one important factor contributing to heterogeneity in sporadic AD, as non-ε4 status among EOAD patients correlates with atypicality [118].

## Conclusion

This review provides a preliminary summary of the various neuroimaging techniques that have been explored for in vivo imaging of AD. MRI and PET imaging are still the mainly used technique in the detection, assessment of AD. From a scientific perspective, more and more multimodality and multiparametric neuroimaging techniques should improve our understanding of brain function and open new insights into the pathophysiology of AD. We expect exciting results will emerge from new neuroimaging applications that will provide scientific and medical benefits.
